# Expressing anti-HIV VRC01 antibody using the murine IgG1 secretion signal in *Pichia pastoris*

**DOI:** 10.1186/s13568-017-0372-7

**Published:** 2017-03-24

**Authors:** Rochelle Aw, Paul F. McKay, Robin J. Shattock, Karen M. Polizzi

**Affiliations:** 1 Department of Life Sciences, Imperial College London, London, SW7 2AZ UK; 2 Centre for Synthetic Biology and Innovation, Imperial College London, London, SW7 2AZ UK; 3 Department of Infectious Diseases, Imperial College London, London, W2 1PG UK

**Keywords:** *Pichia pastoris*/*Komagataella phaffi*, Broadly neutralising antibody, VRC01, Murine IgG1 signal peptide, Alpha-mating factor signal peptide

## Abstract

The use of the recombinant expression platform *Pichia pastoris* to produce pharmaceutically important proteins has been investigated over the past 30 years. Compared to mammalian cultures, expression in *P. pastoris* is cheaper and faster, potentially leading to decreased costs and process development times. Product yields depend on a number of factors including the secretion signal chosen for expression, which can influence the host cell response to recombinant protein production. VRC01, a broadly neutralising anti-HIV antibody, was expressed in *P. pastoris*, using the methanol inducible *AOX1* promoter for both the heavy and light chains. Titre reached up to 3.05 μg mL^−1^ in small scale expression. VRC01 was expressed using both the α-mating factor signal peptide from *Saccharomyces cerevisiae* and the murine IgG1 signal peptide. Surprisingly, using the murine IgG1 signal peptide resulted in higher yield of antibody capable of binding gp140 antigen. Furthermore, we evaluated levels of secretory stress compared to the untransformed wild-type strain and show a reduced level of secretory stress in the murine IgG1 signal peptide strains versus those containing the α-MF signal peptide. As bottlenecks in the secretory pathway are often the limiting factor in protein secretion, reduced levels of secretory stress and the higher yield of functional antibody suggest the murine IgG1 signal peptide may lead to better protein folding and secretion. This work indicates the possibilities for utilising the murine IgG1 signal peptide for a range of antibodies, resulting in high yields and reduced cellular stress.

**Electronic supplementary material:**

The online version of this article (doi:10.1186/s13568-017-0372-7) contains supplementary material, which is available to authorized users.

## Introduction

Over the last 30 years the methylotrophic yeast *Pichia pastoris* has been used to produce heterologous recombinant proteins with over 5000 different proteins expressed to date (Ahmad et al. 2014). The engineering of a humanized glycosylation strain has meant that in addition to enzymes and basic proteins, more complex proteins can be produced with increased efficacy over alternative yeast recombinant expression systems such as *Saccharomyces cerevisiae* (Jacobs et al. 2009; Hamilton and Gerngross 2007). Some such proteins are antibodies, which require the correct glycosylation patterns in order to fold and function correctly.

The anti-HIV broadly neutralising antibody (bNAb), VRC01, has been used in a Phase I clinical trial ending August 2015 (NIAID 2013) and in April 2016 two phase II clinical trials (the AMP studies) were announced to evaluate the safety and efficacy of bNAb VRC01 in preventing HIV-1 infection in high-risk, HIV uninfected women and men and transgender people who have sex with men (NIAID 2015, 2016). Most commonly expressed in human embryonic kidney cells (HEK), this bNAb was originally isolated from patient serum. It neutralizes approximately 90% of circulating viral isolates at IC_50_ of 50 μg mL^−1^, although this value falls to 70% at 1 μg mL^−1^ (Wu et al. 2010). VRC01 actively binds to the HIV envelope glycoprotein gp140, blocking its interaction with CD4+ receptor on T cells and is particularly effective as it directly mimics the structure of the CD4+ receptor (Li et al. 2011).

There are several advantages of using *P. pastoris* as an expression host. It grows to very high cell densities, leading to exceptionally high volumetric productivity. In addition, *P. pastoris* secretes very few native proteins, making downstream processing of secreted products significantly easier. In most instances proteins are fused to the α-mating factor (α-MF) signal peptide from *S. cerevisiae* to target proteins to the secretory pathway. However, there has been extensive research indicating that this is not always the best option (Lin-Cereghino et al. 2013; Damasceno et al. 2012) and optimisation of secretion signals can significantly increase the titre obtained. Native signal peptides from the protein of interest have also been used successfully, such as when expressing the major urinary protein complex (MUP) (Ferrari et al. 1997; Stadlmayr et al. 2010). Other secretion signals are regularly utilised, including non-fungal signals such as the human serum albumin signal peptide, which has been shown to be effective when producing human lysozyme (Xiong and Chen 2008).

Here we evaluated expression with the murine IgG1 signal peptide, which is often used for protein secretion in mammalian cells (Kabanova et al. 2014; Zuo et al. 2000) and has been successfully transferred to antibody expression in *Leishmania tarentolae* and plant based systems (Klatt and Konthur 2012; Chen et al. 2015). Active VRC01 antibody was successfully produced in *P. pastoris* and the murine IgG1 signal peptide resulted in higher secretion yields than using the α-MF signal peptide. As the secretory pathway has often been attributed as the bottleneck when secreting proteins (Love et al. 2012; Ferrer-Miralles et al. 2009; Delic et al. 2013; Mattanovich et al. 2004), it was of interest that strains expressing VRC01 with the murine IgG1 signal peptide show reduced secretory stress, indicating that this may be preferential for the secretion of VRC01 and possibly for antibodies in general.

## Materials and methods

### Media and growth conditions

Bacterial strains were cultured in Lennox lysogeny broth (LB) medium (1% peptone au casein, 0.5% yeast extract, 0.5% NaCl) and supplemented with either 100 μg mL^−1^ Zeocin™ (Thermo Fisher Scientific, Paisley, UK) or 100 μg mL^−1^ ampicillin (Sigma Aldrich, Dorset, UK). Yeast strains were cultured in a rich YPD medium (2% peptone au casein, 1% yeast extract, 2% dextrose). Selection was carried out using Zeocin and Nourseothricin (clonNAT; Jena Bioscience, Jena, Germany) at 100 μg mL^−1^ each. Expression was carried out in buffered glycerol/methanol-complex medium (BMGY/BMMY; 1% yeast extract, 2% peptone, 100 mM potassium phosphate, pH 6.0, 1.34% yeast nitrogen base, 4 × 10^−5^% d-Biotin, 1% glycerol or 0.5% methanol).

### Strain construction

Bacterial recombinant DNA manipulation was carried out in *Escherichia coli* strain NEB 5-α (New England Biolabs, Hertfordshire, UK). The VRC01 heavy and light chain genes were generated from their published sequence (Wu et al. 2010) and cloned into a pUC19 vector already containing the murine IgG1 secretion signal (Tiller et al. 2008). The amino acid sequence of the murine IgG1 secretion signal is GWSCIILFLVATATGVHSQ. The four vectors used in this study were constructed using the DNA Gibson Assembly method (Gibson et al. 2009). Initially the VRC01 heavy and light chains were amplified by PCR using Phusion^®^ High-Fidelity DNA polymerase (New England Biolabs) ensuring that the murine IgG1 secretion signal was included and primers designed to add regions of homology to the relevant vectors. The VRC01 heavy chain gene was integrated into the pPICZ A vector (Thermo Fisher Scientific). The VRC01 light chain gene was integrated into the pJAN-1 vector (Biogrammatics, Carlsbad, USA). A second set of vectors were made with the initial amplification of VRC01 removing the murine IgG1 secretion signal for integration into the pPICZα A vector and pJAN-s1 vector (both containing the α-MF signal peptide from *S. cerevisiae*), which were also amplified with the relevant regions of homology. The PCR fragments were gel extracted using the Zymoclean™ Gel DNA Recovery kit (Zymo Research Corporation, Irvine, USA). DNA was incubated at 50 °C in equimolar concentrations of 100 ng of DNA with the ingredients of the Gibson master mix (Gibson et al. 2009) and 1 μL was transformed into NEB 5-α competent cells (New England Biolabs).

For cloning into *P. pastoris* 5–10 μg of plasmid DNA was linearised with *Pme*I at a single restriction site within the *AOX1* promoter. The vectors were transformed into cells by electroporation according to recommendations in the *Pichia* Expression manual (Thermo Fisher Scientific) into the *P. pastoris* strain *Δku70* (CBS 12694, CBS-KNAW, Fungal Biodiversity Centre, Utretch, The Netherlands) and grown for 3–5 days at 30 °C on YPD medium containing 100 μg mL^−1^ Zeocin (Thermo Fisher Scientific) and 100 μg mL^−1^ Nourseothricin (Jena Bioscience) to select for double transformants.

### Colony PCR

Colony PCR of *P. pastoris* was performed by boiling a sample of the transformed colony in 20 μL of 20 nM NaOH for 15 min. 2 μL of this solution was used as a template for a PCR reaction using Phire HotStart II as described in the manufacturer’s instructions. Primers for the heavy chain were TCA GAT GAA GAA GCC TGG CGA G and GTC CAC CTT GGT GTT GCT GG and for the light chain TGA CAC AGT CTC CAG GCA CC and CTG TTG AAG CTC TTT GTG ACG G.

### Protein expression

Screening expression in *P. pastoris* was performed in 24 deep-well plates in 3 mL of medium and sealed with Breathe-Easy^®^ sealing membrane (Sigma Aldrich). Flask expression in *P. pastoris* was performed in 500 mL glass baffled flasks in 50 mL of medium. Cells were incubated at 30 °C, 216 rpm for 24 h in BMGY to allow growth before being centrifuged at 4000 rpm for 5 min. The supernatant was removed and the medium replaced with BMMY to induce expression. Cultures were left to express at 20 °C, 216 rpm for 72 h before being harvested. Every 24 h the culture was supplemented with 0.5% (v/v) methanol.

### Dot blot analysis

The Bio-Dot^®^ Apparatus (Bio-Rad) was used according to manufacturer’s instructions. 50 μL of sample supernatant from the deep-well plate expression was loaded into individual wells. A vacuum was applied until all the liquid had passed through the membrane. The VRC01 antibody was detected by using a 1:2000 dilution of an AP-conjugated Rabbit Anti-Human IgG H&L (Abcam, Cambridge, UK). Spots were developed using the alkaline phosphate substrate BCIP/NBT kit (Thermo Fisher Scientific).

### Protein analysis

Sodium dodecyl sulfate polyacrylamide gel electrophoresis (SDS–PAGE) was performed using 12% mini-PROTEAN^®^ TGX™ precast gels (Bio Rad, Hemel Hempstead, UK). *P. pastoris* culture supernatants were denatured by boiling for 5 min in either reducing or non-reducing SDS sample buffer (0.0625 M Tris–HCl, pH 6.8, 2.3% (w/v) SDS, 10% (w/v) glycerol and 0.01% Bromophenol blue. ± 5% (v/v) β-mercaptoethanol). Molecular weight was estimated by using a prestained protein ladder (10–170 kDa, Thermo Fisher Scientific). Transfer to an Immobilon^®^-FL PVDF membrane (Millpore (U.K) Ltd, Herfordshire, UK) was performed using a Novex^®^ semi-dry blotter (Thermo Fisher Scientific). VRC01 antibody was detected by using a 1:5000 dilution of an AP-conjugated Rabbit Anti-Human IgG H&L (Abcam). Bands were developed using the alkaline phosphate substrate BCIP/NBT kit (Thermo Fisher Scientific).

### Enzyme-linked immunosorbent assay (ELISA)

ELISA was performed on neat supernatant cultures from flask expression. For the total Ig ELISA, Nunc Maxisorp (Thermo Fisher Scientific) high-binding 96-well microplates were coated with 100 μL anti-human Kappa and Lambda light chain mix (Southern Biotech, Birmingham, USA) in PBS at 4 °C overnight. Wells were washed with 0.05% (v/v) Tween in PBS four times, and subsequently blocked with 200 μL 0.05% (v/v) Tween and 1% (v/v) Bovine Serum Albumin (BSA) in PBS for 1 h at 37 °C. To generate a standard curve 50 μL standard antibody in fivefold serial dilution was added to triplicate wells, with a starting concentration of 1000 ng mL^−1^. The plates were then incubated for 1 h at 37 °C, washed, and 100 μL detection antibody added (anti-human IgG HRP; Southern Biotech), diluted to 1:10,000 in 0.05% (v/v) Tween and 1% BSA in PBS. After 1 h incubation at 37 °C wells were washed a further 4 times. 50 μL TMB (KPL Inc, Milford, USA) was added and incubated for 5 min. 50 μL of Stop solution (KPL Inc) was added and the absorbance measured at 450 nm immediately. To determine antigen-specific binding wells were coated with 50 μL CN54-gp140 antigen at 1 μg mL^−1^ in PBS at 4 °C in place of the IgG capture antibodies.

### Copy number analysis

Copy number analysis was performed as previously described (Aw and Polizzi 2016) with minor differences detailed below. Quantitative PCR was run on genomic DNA using KICStart^®^ SYBR^®^ Green qPCR ReadyMix^™^ (Sigma Aldrich) in an Eppendorf Mastercycler^®^ ep realplex quantitative cycler (Eppendorf UK Ltd, Histon, UK). Copy number of the heavy and light chain was calculated using a standard curve against known concentrations of the pPIC-Heavy and pJan-Light plasmids, respectively. Primers for the Heavy chain were AAT CAC AAG CCC AGC AAC AC and GGG CAT GTG TGA GTT TTG TCA C resulting in a 74 bp amplicon. Primers for the Light chain were TGT CTT CAT CTT CCC GCC ATC and ATT CAG CAG GCA CAC AAC AG resulting in a 70 bp amplicon. Cycling conditions were 95 °C for 5 min followed by 40 cycles of 95 °C for 5 s, 58 °C for 15 s and 72 °C for 10 s with a melting curve afterwards to ensure a single product was being measured. Error bars were calculated using a Taylor series expansion, which takes into consideration the error of the standard curve in addition to the deviation between samples (Gerards 1998).

### RT-qPCR

For reverse transcription (RT)-qPCR, RNA was isolated using RiboPure-Yeast Kit according to manufacturer’s instructions (Applied Biosystems, Warrington, UK). Typically 3 × 10^8^ cells were collected, equivalent to 1 mL of culture with OD_600_ of 1. cDNA was prepared using the High-Capacity cDNA Archive Kit (Applied Biosystems, Warrington, UK) according to the manufacturer’s instructions. 1 μg RNA was used in a total reaction volume of 20 μL. RT-qPCR reactions were set up using the 2× SYBR^®^ Green JumpStart Taq Ready Mix (Sigma-Aldrich, Dorset, UK). An Eppendorf Mastercycler^®^ ep *realplex* quantitative cycler was used (Eppendorf UK Ltd, Histon, UK). Data was analysed using the Pfaffl method, based on ΔΔ−Ct (Pfaffl 2001; Livak and Schmittgen 2001) and normalised to *ACT1* as the housekeeping gene. Primers for *ACT1* were GCT TTG TTC CAC CCA TCT GT and TGC ATA CGC TCA GCA ATA CC. Primers for *KAR2* TCA AAG ACG CTG GTG TCA AG and TAT GCG ACA GCT TCA TCT GG, for *PDI* GCC GTT AAA TTC GGT AAG CA and TCA GCT CGG TCA CAT CTT TG. For *HAC1* two primer sets were used, one indicating total *HAC1* TTC TGG AAA ACC ACG TCG TC and TCC AAA TCT GAG ACG GTA CCA C and one indicating spliced *HAC1* ACA GCT CCA TCA GGT TCC ATC and AAT GGT GCT GCA GGA TGA T. All samples were normalised to Δ*ku70* before addition of methanol. Error bars are representative of the Gaussian error propagation of the standard deviation of s[untreated control], s[treated control], s[untreated sample] and s[treated sample] (Yuan et al. 2006). All statistical analysis was conducted in R (R Core Team 2015). The student *t* test was applied with significant variance observed where p ≤ 0.05.

## Results

### Construction of the VRC01 expression strains

**Fig. 1:**
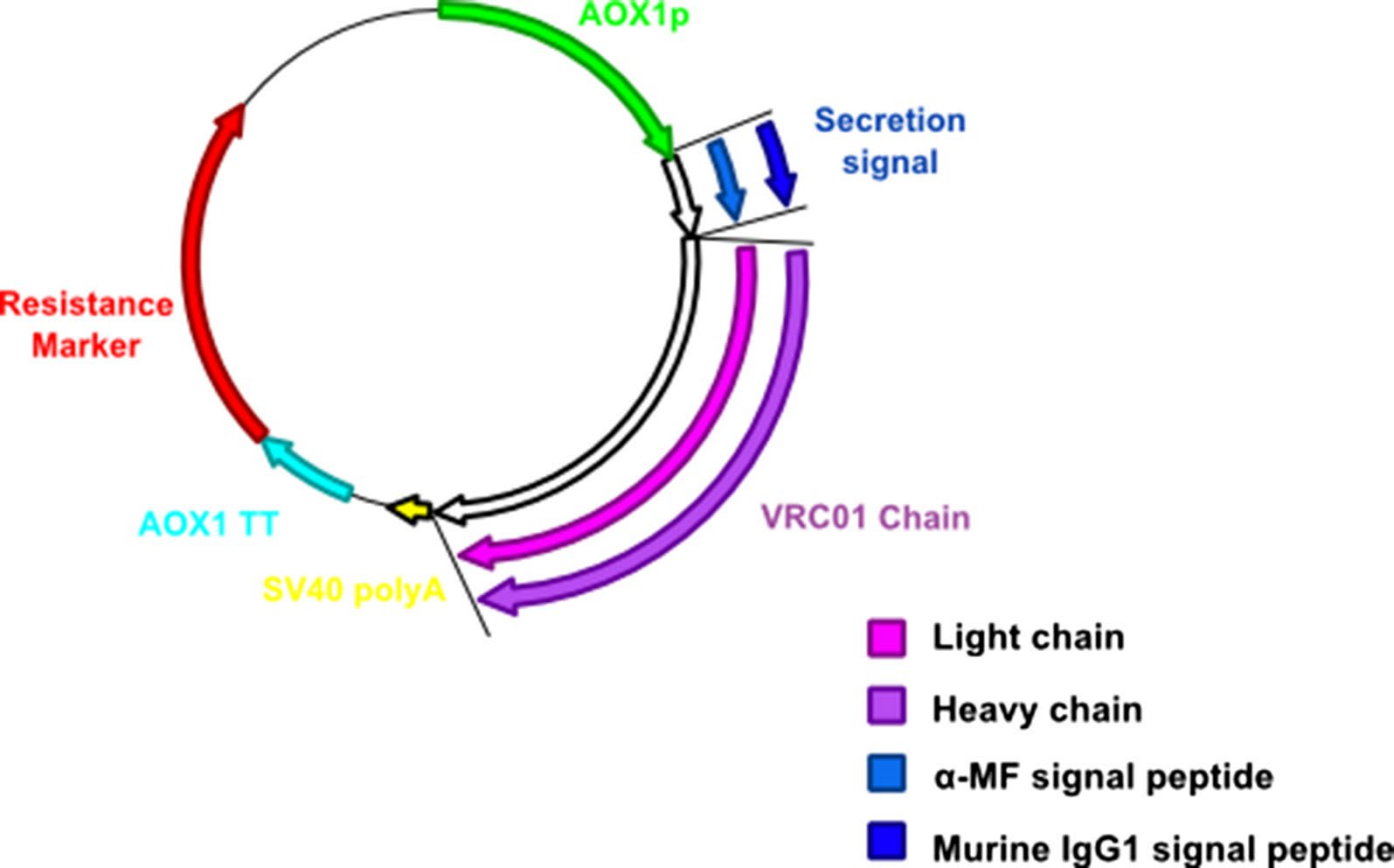
Graphical representation of vector generation. Four vectors were generated containing a combination of either the VRC01 heavy chain or light chain and either the α-MF or murine IgG1 signal peptide. All vectors utilised the *AOX1* promoter for expression

To assess the production of the bNAb VRC01 in *P. pastoris* the heavy and light chains were cloned independently into the commercial vectors pPICZα and pJANs-1, respectively. The α-MF signal peptide is the standard sequence used for extracellular expression and is included in many of the commercial expression vectors available. Therefore, we created vectors where both the heavy and light chain genes were fused to the sequence of the α-MF signal peptide. Additionally, the murine IgG1 signal peptide, which is a popular choice in mammalian expression systems, was also tested. In total four vectors were generated with a combination of either heavy/light chain and α-MF/murine signal peptide (Fig. 1). All VRC01 antibody chains were expressed using the alcohol oxidase 1 (*AOX1*) promoter.

Vectors were transformed into Δ*ku70 P. pastoris* using co-transformation and dual selection on both 100 μg mL^−1^ cloNAT and 100 μg mL^−1^ Zeocin to integrate both the heavy and the light chains. Both vectors integrate into the same locus (*AOX1*), and therefore by using Δ*ku70*, which prevents off-target integration, a higher proportion of clones with the correct integration should be observed (Naatsaari et al. 2014). Individual colonies were selected and the presence of both the heavy and light chains within the *P. pastoris* genome was confirmed by colony PCR (Additional file 1: Figure S1). Clones containing the α-MF signal peptide are referred to as α and those containing the murine IgG1 signal peptide are referred to as M in the subsequent sections.

### Expression of VRC01 antibody in *P. pastoris*

**Fig. 2:**
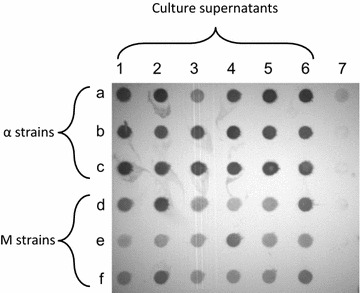
Expression of VRC01 light and heavy chain in *P. pastoris* determined by *dot blot*. *Dot blot* was used to confirm the expression of the VRC01 from culture supernatants using a Rabbit Anti-Human IgG heavy and light antibody conjugated to alkaline phosphatase. *1*–*6* culture supernatants; *a*–*c*—α-MF strains, *d*–*f*—murine IgG1 strains. *7a* Δ*ku70*, *7b*–*f* blank

To analyse expression, 18 single colonies from both α and M transformations were inoculated in 3 mL of BMGY in 24 deep-well plates and grown for 24 h in BMGY prior to induction using the methanol containing medium BMMY. Wild-type Δ*ku70* was subjected to the expression conditions and analysed as a control. Cultures were left to express for 72 h at 20 °C due to the complexity of the protein to aid folding and assembly. Dot blot analysis was used to determine qualitatively whether the VRC01 was expressed into the supernatant (Fig. 2). The dot blot results show that on average supernatant from the α strains contains a higher concentration of VRC01 antibody than the M strains.

Based on the dot blot results, two clones that expressed a high level of the VRC01 antibody were selected from both the α and M strains for expression in shake flasks. Copy number analysis was carried out to ensure the strains analysed were of similar copy number (Additional file 2: Figure S2). α1 has one copy of both the heavy and light chains, while α2 and M1 have 2 copies of the heavy chain and 1 of the light chain and M2 has two copies of both the heavy and light chains (Additional file 2: Figure S2). The four strains were inoculated into 50 mL BMGY and grown for 24 h shaking at 30 °C. After 24 h, the culture supernatant was removed, and the medium replaced with BMMY. Cultures were expressed for a total of 72 h, feeding an additional 0.5% (v/v) methanol every 24 h. At 0, 2, 4 and 6 RNA samples were collected and after 72 h the supernatant was collected for analysis.

**Fig. 3:**
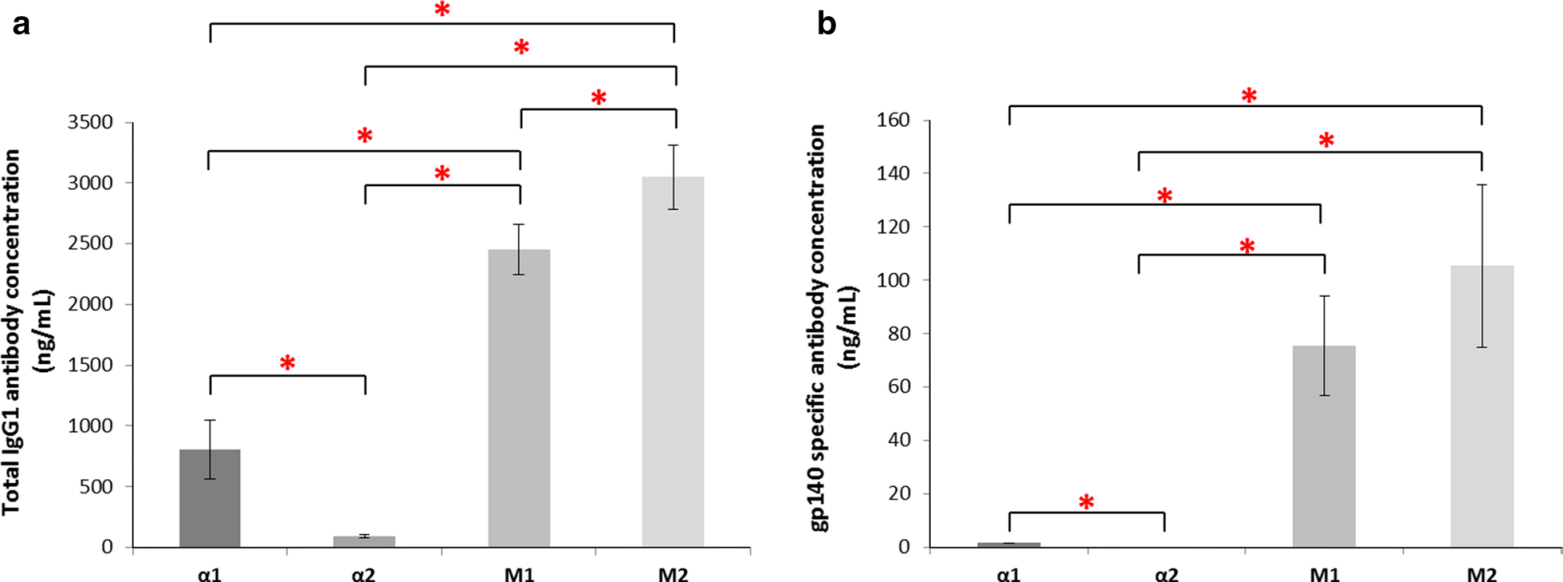
Concentration of total antibody and gp140 specific antibody. ELISA was used to determine concentration of total IgG1 antibody and gp140 specific antibody (n = 3). **a** Total IgG1 antibody. **b** gp140 specific antibody

The dot blot is a qualitative analysis, which does not give an indication of whether the protein is correctly folded or functional. Therefore, ELISA was undertaken to quantify the yield of intact antibody (Fig. 3) and Western blots were performed under both reducing and non-reducing conditions to observe the ratio of heavy and light fragments (Additional file 3: Figure S3). The ELISA procedures for both total IgG1 and gp140 binding antibody concentration measurements require both the heavy and light chain to be expressed for a signal to be detected, unlike the dot blot which only requires either of the chains to be present. Total IgG1 antibody yield (Fig. 3a) ranged from 0.09 (α2) to 3.05 μg mL^−1^ (M2). The M1 and M2 strains showed significantly higher total antibody yield (2.45 and 3.05 μg mL^−1^, respectively) than the α1 and α2 strains (0.81 and 0.09 μg mL^−1^, respectively). The discrepancy between the ELISA measurements and the dot blot results could be the result of an imbalance between the amount of light and heavy chain secretion as it has been previously shown that light chain can be secreted on its own (Gasser et al. 2006; Gach et al. 2007; Lee et al. 1999). Since the copy number of α2 and M2 are the same (Additional file 2: Figure S2) the differences in yield cannot be a result of differences in copy number.

It was noticeable that within the subset groups (α and M), α1 shows significantly higher (p = 0.0068) titre compared to α2, despite having lower copy number of the heavy chain (Additional file 2: Figure S2). However, M2 showed significantly higher yield (p = 0.0373) than M1, and in this instance M2 had the higher copy number. This implies that having more than one copy of each of the genes for heavy and light chains does not cause secretion saturation (Hohenblum et al. 2004) and the differences in yield observed cannot be accounted for by copy number alone. Despite the individual differences among the α and M strains, the strains containing the murine IgG1 signal peptide had a volumetric productivity three times higher the best strain utilising the α-MF signal peptide.

From the Western blot results it is clear that α2 shows significantly less expression than the other strains (Additional file 3: Figure S3). Furthermore, the non-reducing gel (Additional file 3: Figure S3b) shows a strong signal at 55 KDa, particularly in the α1 strain, which could be excess heavy chain or dimerized light chain. This may explain the reduced yields in the ELISA results (Additional file 2: Figure S2), despite the strong signal from the dot blot (Fig. 2).

Additionally, an ELISA was run using the gp140 antigen, giving an indication of active antibody (Fig. 3b). Here the results between M1 and M2 were not significantly different (p = 0.219) with M1 at 75.489 ng mL^−1^ and M2 at 105.32 ng mL^−1^. Levels of gp140 specific antibody from α2 were undetectable, indicating there may have been a problem with the folding and/or secretion of the antibody in this specific strain. Levels of antibody capable of binding to gp140 for α1 are also significantly lower than both M1 and M2 (p = 0.0300 and 0.0401, respectively) at 1.53 ng mL^−1^. Therefore, in addition to higher total antibody concentration, specific antigen binding antibody titres were higher for both murine IgG1 signal peptide strains.

### UPR signalling and secretory stress

**Fig. 4:**
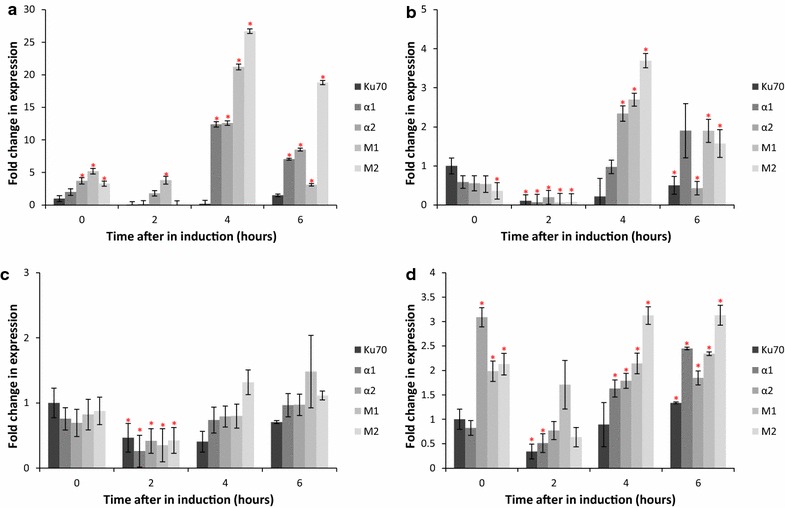
Changes in gene expression for elements of the UPR for VRC01 expressing strains. RT-qPCR was used to determine gene expression for *KAR2*, *PDI¸* total *HAC1* and spliced *HAC1*. *ACT1* was used as the housekeeping gene. Fold change was calculated using the Pfaffl calculation, as a comparison to Δ*ku70* 0 h. *Error bars* are a representation of Gaussian error propagation from three technical replicates of three biological replicates. *Red asterisks* indicate a significant difference compared to Δ*ku70* at 0 h, where p ≤ 0.05. **a** Spliced *HAC1*; **b** *KAR2*; **c** *PDI1*; **d** Total *HAC1*

To ascertain whether the differences in yield between strains were due to secretory stress, RT-qPCR was used to analyse gene expression levels of UPR related genes. We analysed *KAR2*, *PDI1* and *HAC1* looking at both total *HAC1* and spliced *HAC1.* Samples were taken at 0 h (pre-induction) and then 2, 4 and 6 h post-induction. All samples were compared to the untransformed Δ*ku70* wild-type strain at 0 h (Fig. 4a–d).

After induction with methanol all strains show upregulation of the UPR markers investigated except for *PDI1*. This includes upregulation of UPR markers in the wild-type strain, which has been previously observed as a result of the switch to methanol, which requires large-scale cellular reorganisation to produce the required peroxisomes (Vanz et al. 2012; Edwards-Jones et al. 2015).

*HAC1* splicing (Fig. 4a) is commonly used as an indicator of UPR induction, as the spliced variant is translated into the transcription factor responsible for UPR propagation after binding the UPR elements (UPRE) (Guerfal et al. 2010). From 4 h, all strains expressing VRC01 show consistently higher levels of spliced *HAC1* suggesting rapid UPR activation. Interestingly, at 4 h M1 and M2 show a higher degree of *HAC1* splicing than α1 and α2, suggesting some UPR response is beneficial to cells (Gasser et al. 2008).

Spliced *HAC1* RNA is translated into a functional transcription factor, which activates the expression of UPR target genes include the folding chaperones *KAR2* and *PDI1* (Mattanovich et al. 2004). In addition, the promoter region of *HAC1* also contains UPRE and therefore total *HAC1* levels are also an indicator of UPR signalling (Guerfal et al. 2010). For *KAR2* (Fig. 3b) after 4 h there appears to be a significant upregulation in α2, M1 and M2 compared to the wild-type strain. The α1 strain does not show significant upregulation of *KAR2*, although RNA levels are elevated at 6 h. This suggests that α1 has a lower UPR response than the other three strains, which may be due to the fact that it has single copies of the heavy and light chain genes.

Interestingly, for *PDI1* expression (Fig. 4c), at 2 h all strains showed a significant downregulation compared to Δ*ku70* at 0 h, a trend that was also observed for *KAR2* expression. Although co-expression of chaperones such as *PDI1* along with a protein of interest has often been shown to have mixed results in improving recombinant protein yield (Inan et al. 2006; Aw and Polizzi 2013), some *PDI1* activation would be expected because the promoter region contains UPRE. While the lack of upregulation is puzzling, the fact that the other UPR markers, including spliced *HAC1* are upregulated, strongly indicates that the UPR is activated.

As mentioned above, total *HAC1* levels can also be considered a marker of UPR. Even before induction total *HAC1* levels in α2, M1 and M2 showed significantly higher expression compared to Δ*ku70* at 0 h (Fig. 4d). The *AOX1* promoter is not known to have leaky expression when grown in shake flasks (Aw and Polizzi 2016), even though this is not the case for bioreactors (Bawa et al. 2014). Therefore, this implies that there may be other stresses on α2, M1 and M2, such as the onset of starvation. Interestingly, 2 h after induction with methanol, levels of total *HAC1* return to baseline. However, after 4 h of expression all the strains producing VRC01 once again show a significant upregulation of total *HAC1* levels compared to wild-type. Total *HAC1* levels remain elevated in all strains, except for α1.

Based on the total profile of UPR marker expression, the α2 strain shows the highest level of stress, suggesting that the low antibody yield from this strain might be due to a bottleneck in the secretory pathway. This correlates with the low levels of expression observed with ELISA for both total IgG1 and gp140 binding antibody. The copy number for α2 is the same as that for M1, which implies that the differences in volumetric productivity and secretory stress are not due to copy number or a result of secretion saturation. Furthermore, while the light chain is capable of being secreted on its own, the heavy chain must be complexed with the light chain for secretion (Gasser et al. 2006; Gach et al. 2007; Lee et al. 1999). Therefore, the limiting factor in intact antibody yield is the amount of heavy chain, which should theoretically be produced to higher amounts in α2 and M1 compared to α1 since two copies of the heavy chain have integrated.

For α1, on the other hand, the low yield could be explained by the fact that this is the only strain with a single copy of the heavy chain. In addition, there is a distinct lack of UPR upregulation. Although moderate levels of *HAC1* splicing are observed 4 h after induction, this does not result in strong upregulation of chaperones or total *HAC1* levels. It is possible that the levels of antibody produced by α1 are insufficient to activate UPR. Alternatively, since the effects of UPR are known to have a positive influence on protein yield (Gasser et al. 2008), the weak induction of UPR may perhaps explain the lower yield from α1 compared to the M1 and M2 strains.

## Discussion

We have demonstrated successful expression of the bNAb VRC01 using *P. pastoris* as an expression platform with a signal sequence commonly used in mammalian expression vectors. It is the authors understanding that this is the first instance where the murine IgG1 signal peptide, which is commonly used in mammalian antibody expression vectors, has been used in *P. pastoris*. Although total volumetric productivities were not as high as has been reported for other full length antibodies (Ye et al. 2011), expression was carried out in shake flasks, and not bioreactors, and no growth optimisation was performed. Shah et al. recently described a high throughput method for constructing strains that express a range of anti-HIV bNAbs, with yields were between 100 and 500 ng mL^−1^, comparable to those shown here (Shah et al. 2015). Gasser et al. saw no expression of either Fab or ScFV utilising the human secretion signal from 3D6 light chain (Gasser et al. 2006). However, they also were not able to detect any antibody expression using the *AOX1* promoter, suggesting that differences in sequence or format may be the causes of differences.

Overall, the trends in the total and gp140 binding antibody yields from the ELISA data are comparable, i.e., strains with higher total antibody expression also have higher yields of specific binding antibody. The lower apparent yield of the antibody from the antigen-specific ELISA is likely due to different binding affinities of the antibody to the plate bound antigen in contrast to the much higher affinity capture ELISA used to detect total Ig. The plate bound antigen is distorted due to electrostatic stresses, leading to a reduction in the ability of the produced antibody to bind. Our results here with the antibody expressed in *P. pastoris* are identical to the binding seen with the same antibody produced in mammalian 293T cells, where the apparent antigen-specific yield is 1/20th–1/25th of that measured for the total Ig present (Additional file 4: Table S1).

The dot blot indicated that higher average yields were achieved in strains using the α-MF signal peptide. However, the ELISA results indicated that M1 and M2, which utilised the murine IgG1 signal peptide to direct protein to the secretory pathway, produced significantly higher yields of antibody than both the α1 and α2 strains. The discrepancy between the dot blot and ELISA results may be due to differences in the analytical techniques. The antibody used in the dot blot detects both light and heavy chains and therefore cannot distinguish between individual chains and intact antibody. Previous work has reported that the light chain can be secreted without dimerization to the heavy chain (Gasser et al. 2006; Gach et al. 2007; Lee et al. 1999), which can confound the dot blot results. In our total IgG ELISA procedure both the heavy and the light chain will be required to produce a signal because the antibodies are captured using antibodies that bind to the light chain and detected using antibodies that bind the heavy chain thus ensuring both parts are present. In addition, the gp140 specific ELISA will require the correct conformation of the antibody as it captures based on antigen binding. This was supported by the Western blot analysis that indicates an imbalance of heavy and light chain between the strains (Additional file 3: Figure S3).

There have been previous reported instances where utilising the α-MF as a signal sequence has not resulted in the highest yield (Lin-Cereghino et al. 2013; Ferrari et al. 1997; Heiss et al. 2015; Fitzgerald and Glick 2014). This phenomenon seems to be protein-dependent and different options are often examined during strain optimisation. Our results suggest that the murine IgG1 signal peptide should be included among those options as it can be successfully utilised by *P. pastoris* to direct proteins to the secretory pathway.

The α-MF signal peptide from both the pPICZα and pJANs-1 vector contains EAEA repeats targeted for Ste13 cleavage (Brake et al. 1984). It has previously been reported that removing this repeat region results in an increased homogeneity of N-terminal sequence of secreted proteins; however yields are reduced (Tanghe et al. 2015; Kozlov and Yagudin 2008). Future analysis of the N-terminus of the antibody produced here may provide insights into the processing of the murine IgG1 signal peptide, which does not have EA repeats and therefore should not be a substrate for STE13.

It would be interesting to determine whether the benefits of using the murine IgG1 signal peptide reported here extend to monocistronic expression vectors. Both Gasser et al. and Burtet et al. used a flexible linker to join the fragments to prevent uncoupled light chain from being secreted independently of the heavy chain (Burtet et al. 2007; Gasser et al. 2006). Alternatively, a 2A sequence, which allows for multiple genes to be expressed simultaneously under the same promoter, could be used (Kim et al. 2011; Shah et al. 2015; Geier et al. 2015). The 2A peptide functions co-translationally by causing a ribosome skip that cleaves between the glycine and final proline (de Felipe et al. 2006; Donnelly et al. 2001; Trichas et al. 2008) to produce separate polypeptide chains. Utilising a 2A sequence allows for more efficient processing of the genes both upstream and downstream of the sequence and may also result in a reduced burden to the cell, which may be beneficial for productivity and could improve the ratio of expression between the light and heavy chains (Shah et al. 2015; Geier et al. 2015; Burtet et al. 2007).

In order to establish whether secretory stress on the strains resulted in differences of expression between those containing the α-MF signal peptide and those containing the murine IgG1 signal peptide transcript levels of indicators of the UPR were evaluated. It was evident that the α2 strain was inherently sick, showing significant evidence of UPR signalling. The α1 strain, conversely, showed signs of a failure to upregulate UPR sufficiently to return to homeostasis, although this may be due to the single copy of the heavy chain integrated into the genome. Therefore, different underlying biochemistry is responsible for the lower yield in these two strains. The lower yields of the α strains may be a result of clonal variation, a well-established phenomenon in *P. pastoris* (Cregg et al. 1985; Schwarzhans et al. 2016), and have less to do with the utilisation of the α-MF over the murine IgG1 signal peptide. However, the strong results that we observed for the murine IgG1 signal peptide do indicate that achieving high yields are possible.

In summary, we have successfully expressed the bNAb VRC01 using *P. pastoris* and have shown that it is possible to utilise both the α-MF signal peptide and the murine IgG1 signal peptide for secretion. The highest concentration of total antibody obtained was 3.05 μg mL^−1^. Interestingly, the levels of secretory stress appear reduced in the strains containing the murine IgG1 signal peptide and volumetric productivity was significantly increased for both strains compared to those utilising the α-MF signal peptide. Therefore, in the future, it would be useful to include the murine IgG1 signal peptide in expression optimisation experiments for antibodies, which could improve yield.

## Additional files

**Additional file 1: Figure S1.** Confirmation of integration of heavy and light chains into *P. pastoris*. Colony PCR was used to confirm integration of both the independent light and heavy chains into *P. pastoris.*

**Additional file 2: Figure S2.** Copy number of heavy and light chains of α1, α2, M1 and M2. Copy number was determined by qPCR using a standard curve from plasmid DNA. Error bars are calculated using a Taylor series expansion for error propagation.

**Additional file 3: Figure S3.** Expression of VRC01 light and heavy chain in *P. pastoris* determined by Western Blot. Western blot was used to confirm the expression of both the light and heavy chains on a denaturing gel, using a secondary Rabbit Anti-Human IgG heavy and light antibody in both reducing and non-reducing conditions. **A:** reducing conditions; **B:** non-reducing conditions.

**Additional file 4: Table S1.** Calculated values for gp140-specific VRC01 capture against starting concentration of VRC01. The results show 4–5% (1/20 to 1/25) binding capabilities.

## Abbreviations

α-MF: α-mating factor signal peptide
α: α-mating factor signal peptide strains
α1: *P. pastoris* expressing strain of VRC01 using the α-mating factor signal peptide
α2: *P. pastoris* expressing strain of VRC01 using the α-mating factor signal peptide
*AOX1*: alcohol oxidase 1
BMGY: buffered-complex glycerol media
BMMY: buffered-complex methanol media
bNAbs: broadly neutralising antibodies
IgG: immunoglobulin G
M: murine IgG1 signal peptide strains
M1: *P. pastoris* expressing strain of VRC01 using the murine IgG1 signal peptide
M2: *P. pastoris* expressing strain of VRC01 using the murine IgG1 signal peptide
UPR: unfolded protein response
UPRE: unfolded protein response element
