# Supplementary figures and images for: Expressing anti-HIV VRC01 antibody using the murine IgG1 secretion signal in Pichia pastoris
Source: AMB Express. 2017 Mar 24;7:70. doi: 10.1186/s13568-017-0372-7 (PMC5366992; doi:10.1186/s13568-017-0372-7)

# Heavy Chain

# Light Chain

KB

L

$\alpha 1$

$\alpha 2$

M1

M2

L

$\alpha 1$

$\alpha 2$

M1

M2

2000

1500

1000

800

600

400

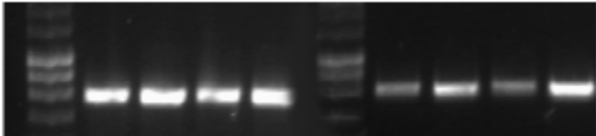

Supplement: Supplementary file 1 — Additional file 1: Figure S1. Confirmation of integration of heavy and light chains into P. pastoris. Colony PCR was used to confirm integration of both the independent light and heavy chains into P. pastoris. [file 13568_2017_372_MOESM1_ESM.pdf]

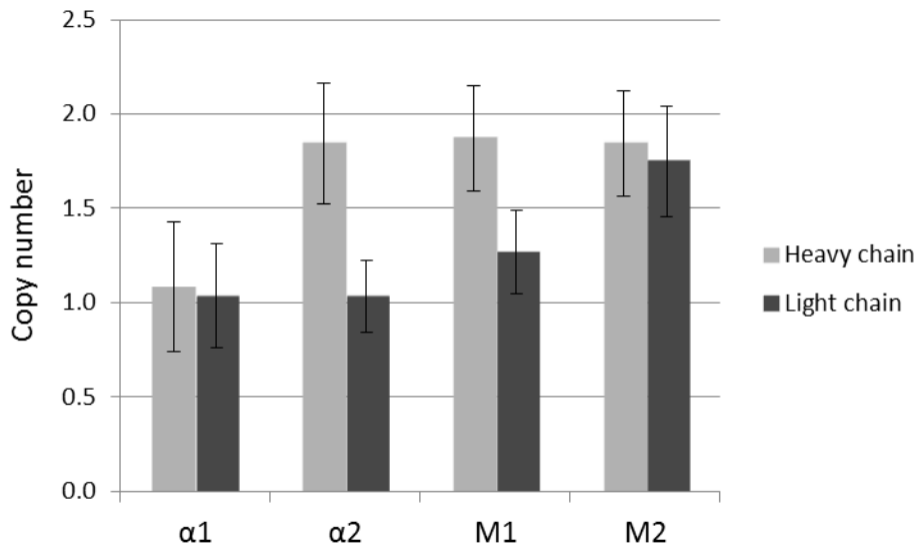

Supplement: Supplementary file 2 — Additional file 2: Figure S2. Copy number of heavy and light chains of α1, α2, M1 and M2. Copy number was determined by qPCR using a standard curve from plasmid DNA. Error bars are calculated using a Taylor series expansion for error propagation. [file 13568_2017_372_MOESM2_ESM.pdf]

**A**L       $\alpha 1$        $\alpha 2$       M1      M2180  
130  
100  
75  
55  
40  
35  
25  
15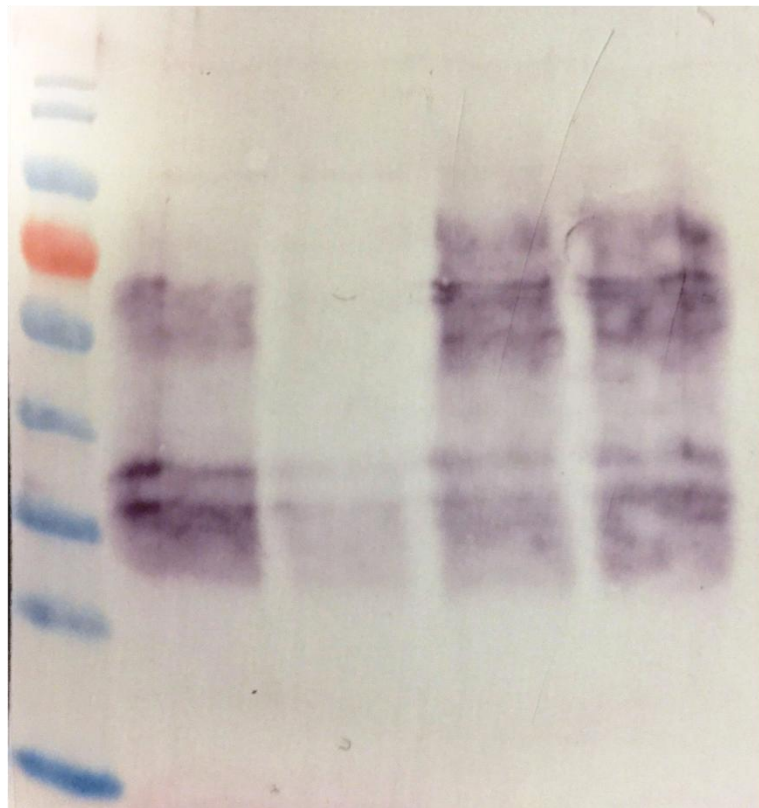**B**L       $\alpha 1$        $\alpha 2$       M1      M2180  
130  
100  
75  
55  
40  
35  
25  
15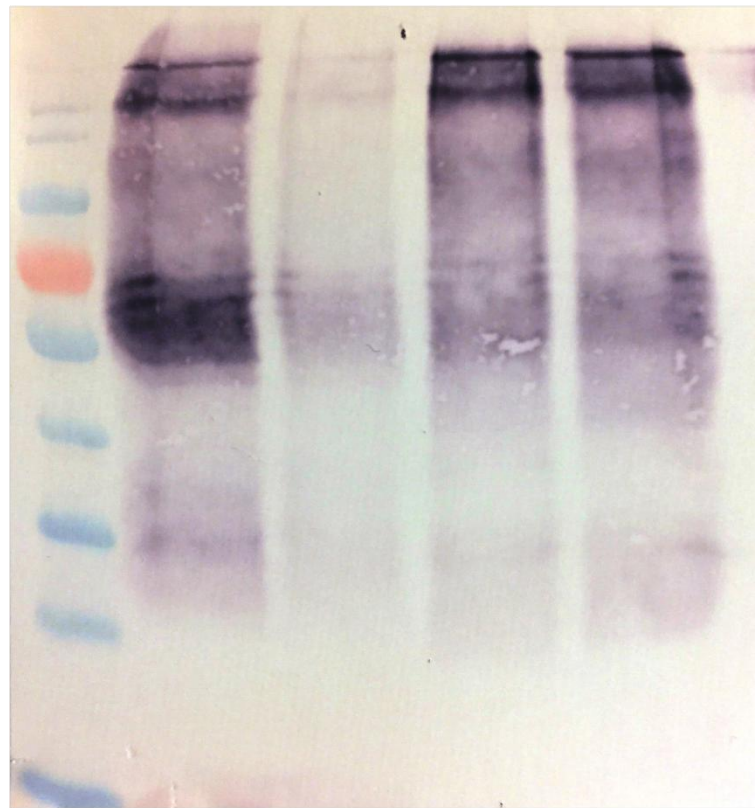

Supplement: Supplementary file 3 — Additional file 3: Figure S3. Expression of VRC01 light and heavy chain in P. pastoris determined by Western Blot. Western blot was used to confirm the expression of both the light and heavy chains on a denaturing gel, using a secondary Rabbit Anti-Human IgG heavy and light antibody in both reducing and non-reducing conditions. A: reducing conditions; B: non-reducing conditions. [file 13568_2017_372_MOESM3_ESM.pdf]
