# Supplementary material for: Expressing anti-HIV VRC01 antibody using the murine IgG1 secretion signal in Pichia pastoris
Source: AMB Express. 2017 Mar 24;7:70. doi: 10.1186/s13568-017-0372-7 (PMC5366992; doi:10.1186/s13568-017-0372-7)
Supplement: Supplementary file 4 — Additional file 4: Table S1. Calculated values for gp140-specific VRC01 capture against starting concentration of VRC01. The results show 4–5% (1/20 to 1/25) binding capabilities. [file 13568_2017_372_MOESM4_ESM.docx]

**Table S1. Calculated values for gp140-specific VRC01 capture against starting concentration of VRC01**. The results show 4-5% (1/20 to 1/25) binding capabilities.

| **VRC01 [ ]** | **Calculated gp140-specific VRC01** |
| --- | --- |
| 1000 ng/ml | 40 ng/ml |
| 200 ng/ml | 10 ng/ml |
